# A Case of Pyometra Due to Plugging of the Cervix by a Fibroid Polypus

**Published:** 1904-04-23

**Authors:** Wheelton Hind

**Affiliations:** Surgeon, North Staffordshire Infirmary.


### Hospital Clinics and Medical Progress.

### A CASE OF PYOMETRA DUE TO PLUGGING OF THE CERVIX BY A FIBROID POLYPUS.

By Wheelton Hind, M.D., B.S., F.R.C.S., Surgeon, North Staffordshire Infirmary.

Pyometra is a somewhat rare condition generally consequent on metritis, with either stricture or occlusion of the os uteri, so that septic discharges cannot drain away. The condition may also arise from the presence of a foreign body such as a portion of a tent, or other article, introduced and accidentally left in the uterus which, while setting up irritation, acts as a cup and ball valve, preventing free drainage. By pyometra may be understood a dilated uterus containing septic matter, as distinguished from metritis, where the interior of the uterus may be septically infected, but there is no damming back of pus. Pyometra may be due to one other condition, which I have not seen mentioned, and that is the presence of a firm uterine polypus which acts as a plug and prevents drainage, of which the following case is a good example.

Mrs. ——, aged 36, admitted to hospital under me on October 31, 1903. She states that she has had a foul copious discharge of pus, which has been increasing in quantity during the last nine months. It has been intermittent in flow, and has become more and more offensive of late. She has had violent metrorrhagia, the periods extending over three weeks. On examination the patient was well nourished and fairly healthy looking, pulse 88 per minute, temperature 100° F. Urine 32 oz. in first 24 hours, sp. gr. 1,010. No albumen. On palpating the abdomen there was a firm, elastic, rounded tumour, reaching up to the umbilicus, which gave a sensation of heat to the hand, and was pyriform in shape; pressure caused an exudation of pus from the vagina. Per vaginam a large polypoid mass was felt, round which the finger could be swept, much offensive pus coming away on manipulation. The tumour rapidly diminished during the night, and when placed on the operating table next day was not easily felt through the abdominal wall. The patient was placed in the lithotomy position, and at once the trouble was found to be due to a large submucous fibroid which, though protruding into the vagina, was tightly grasped above by the cervix. Copious irrigation was carried on for some time, the uterine cavity being thoroughly washed out till all pus was evacuated, then the tumour was removed. Great difficulty was experienced in delivering the growth from the vagina, on account of its size, but once delivered, the pedicle was cut through with scissors. On exploration of the uterus the cavity was found much dilated and the pedicle very short, a condition which no doubt caused the damming back of menstrual hæmorrhage, which, becoming septic, gave rise to the pus. Convalescence was retarded by an attack of pelvic cellulitis, which gave rise to very little general disturbance and cleared up with rest and hot antiseptic douches. All discharge stopped in a few days after the operation, and the uterus soon attained its normal proportions.

Uterine polypi are common enough, but it is very rare that they completely block the cervix, and give rise to pyometra.

